# Comparison of TGSE-BLADE DWI, RESOLVE DWI, and SS-EPI DWI in healthy volunteers and patients after cerebral aneurysm clipping

**DOI:** 10.1038/s41598-022-22760-6

**Published:** 2022-10-21

**Authors:** Sachi Okuchi, Yasutaka Fushimi, Kazumichi Yoshida, Satoshi Nakajima, Akihiko Sakata, Takuya Hinoda, Sayo Otani, Hajime Sagawa, Kun Zhou, Yukihiro Yamao, Masakazu Okawa, Yuji Nakamoto

**Affiliations:** 1 Department of Diagnostic Imaging and Nuclear Medicine, Graduate School of Medicine, Kyoto University, 54 Shogoin Kawaharacho, Sakyoku, Kyoto, 6068507 Japan; 2 Department of Neurosurgery, Graduate School of Medicine, Kyoto University, Kyoto, Japan; 3 Division of Clinical Radiology Service, Kyoto University Hospital, Kyoto, Japan; 4 Siemens Shenzhen Magnetic Resonance Ltd, Shenzhen, China

**Keywords:** Medical research, Neurological disorders

## Abstract

Diffusion-weighted magnetic resonance imaging is prone to have susceptibility artifacts in an inhomogeneous magnetic field. We compared distortion and artifacts among three diffusion acquisition techniques (single-shot echo-planar imaging [SS-EPI DWI], readout-segmented EPI [RESOLVE DWI], and 2D turbo gradient- and spin-echo diffusion-weighted imaging with non-Cartesian BLADE trajectory [TGSE-BLADE DWI]) in healthy volunteers and in patients with a cerebral aneurysm clip. Seventeen healthy volunteers and 20 patients who had undergone surgical cerebral aneurysm clipping were prospectively enrolled. SS-EPI DWI, RESOLVE DWI, and TGSE-BLADE DWI of the brain were performed using 3 T scanners. Distortion was the least in TGSE-BLADE DWI, and lower in RESOLVE DWI than SS-EPI DWI near air–bone interfaces in healthy volunteers (*P* < 0.001). Length of clip-induced artifact and distortion near the metal clip were the least in TGSE-BLADE DWI, and lower in RESOLVE DWI than SS-EPI DWI (*P* < 0.01). Image quality scores for geometric distortion, susceptibility artifacts, and overall image quality in both healthy volunteers and patients were the best in TGSE-BLADE DWI, and better in RESOLVE DWI than SS-EPI DWI (*P* < 0.001). Among the three DWI sequences, image quality was the best in TGSE-BLADE DWI in terms of distortion and artifacts, in both healthy volunteers and patients with an aneurysm clip.

## Introduction

Diffusion-weighted imaging (DWI) is an essential MRI technique for assessing neurological disorders^1^. Single-shot echo-planar imaging (SS-EPI) is the most widely used DWI technique; however, EPI-based DWI techniques are prone to susceptibility artifacts in areas of B_0_ field inhomogeneity, such as near air–bone interfaces and near metallic implants^2^.

Many techniques have been developed for DWI to overcome distortion caused by magnetic susceptibility. These include field mapping corrections^3,4^, multi-shot EPI^5,6^, readout-segmented EPI (RESOLVE DWI)^7–9^, point-spread function (PSF) encoding^10,11^, reversed gradient EPI (RG-EPI)^12^, single-shot turbo-spin-echo (SS-TSE)^13^, zoomed EPI^14^, and periodically rotated overlapping parallel lines with enhanced reconstruction (PROPELLER) DWI^15–17^. Each technique has its own advantages and drawbacks that should be considered before application in clinical examinations^3^. RESOLVE DWI reduces geometric distortion, T2* blurring, and susceptibility artifacts through segmented acquisition of k-space along the readout direction, reducing echo spacing and echo time^3,9^.

PROPELLER DWI is a non-EPI DWI technique based on a turbo spin-echo sequence with non-Cartesian BLADE trajectory^18^ that is insensitive to B_0_-related artifacts, and is also termed TSE-BLADE DWI. In addition, it is robust to patient motion because the central k-space is oversampled by radial-like samplings^18^. Nevertheless, the long acquisition time and high specific absorption rate (SAR) prevent its clinical application^19,20^. To overcome these shortcomings, TGSE-BLADE DWI (2D turbo gradient- and spin-echo diffusion-weighted imaging with non-Cartesian BLADE trajectory) has been introduced^21–23^. TGSE-BLADE DWI features multi-blade k-space filling strategy and has a shorter acquisition time than conventional BLADE DWI^21–23^. Moreover, SAR is decreased due to the use of gradient echoes and reduction in the number of refocusing RF pulses. By placing gradient echoes and spin echoes into separate blades and removing off-resonance phases, off-resonance artifacts are reduced^19,20,22,23^.

Several studies have used TGSE-BLADE DWI to reduce geometric distortion and susceptibility artifact in regions where strong magnetic susceptibility presents^20,24–26^. However, it remains unknown whether TGSE-BLADE DWI can reduce clip-induced artifact and improve image quality in patients who have undergone cerebral aneurysm clipping. Acute infarction occasionally occurs after clipping of cerebral aneurysms^27,28^; however, signal intensity dropout and signal pileup caused by clip-induced artifact complicate the diagnosis of acute infarction. To the best of our knowledge, no previous TGSE-BLADE DWI study has performed a quantitative comparison of distortion and artifacts among various intracranial regions in healthy volunteers.

The purpose of this study was to compare distortion and artifacts among three diffusion acquisition techniques (SS-EPI DWI, RESOLVE DWI, and TGSE-BLADE DWI) in healthy volunteers and in patients with a cerebral aneurysm clip.

## Materials and methods

### Phantom study

A phantom was created by embedding one aneurysm clip (Sugita Titanium Aneurysmal Clip II; MIZUHO Corporation) in the center of a cylindrical container filled with agarose gel. This phantom was used to evaluate image distortion in the three DWI sequences.

### Participants

This prospective study was performed in accordance with the Declaration of Helsinki and was approved by Kyoto University Graduate School and Faculty of Medicine, Ethics Committee. Written informed consent was obtained from all participants.

We prospectively enrolled 17 healthy volunteers, and 20 patients who had undergone follow-up MRI between May 2021 and October 2021 after surgical cerebral aneurysm clipping. The exclusion criterion was insufficient image quality due to motion artifacts.

### Image acquisition

MRI was performed using a 3 T scanner (MAGNETOM Skyra or MAGNETOM Prisma; Siemens Healthineers) with a 32-channel head coil or a 64-channel head/neck coil. Three DWI sequences (SS-EPI DWI, RESOLVE DWI, and TGSE-BLADE DWI) were acquired as well as T2-weighted imaging (T2WI).

Table 1 provides the detailed imaging parameters for the three DWI sequences. SS-EPI DWI and RESOLVE DWI are commercially available products, and TGSE-BLADE DWI is a prototype sequence.

**Table 1:** Detailed imaging parameters for SS-EPI DWI, RESOLVE DWI, and TGSE-BLADE DWI.

Parameter	SS-EPI DWI	RESOLVE DWI	TGSE-BLADE DWI
b value (s/mm^2^)	0, 1000	0, 1000	0, 1000
TR (ms)	5000, 5500*	4560, 6240*	7000, 7700*
TE (ms)	53, 77*	55, 64*	46, 61*
FA (degrees)	NA	180	120
FOV(mm)	220 × 220	220 × 220	220 × 220
Phase resolution (percentage)	80	100	NA
Partial Fourier	7/8	Off	Off
Interpolation	On	Off	Off
Matrix	320 × 320	160 × 160	160 × 160
Slice thickness(mm)	3	3	3
Number of slices	35	35	35
Voxel size (mm^3^)	0.7 × 0.7 × 3.0	1.4 × 1.4 × 3.0	1.4 × 1.4 × 3.0
Bandwidth(Hz/pixel)	1202	679	520
NEX	4	1	1
Parallel imaging	GRAPPA 3x	GRAPPA 2x	NA
Turbo factor	NA	NA	13
EPI factor	128	80	3
Readout segments	NA	7	NA
Fat suppression	CHESS	CHESS	CHESS
Acquisition time	1m47s	2m50s, 3m52s*	3m53s, 4m16s*

*Parameters for MAGNETOM Skyra. repetition time, TR; echo time, TE; flip angle, FA; field of view, FOV; number of excitations, NEX; echo planar imaging, EPI; chemical shift selective, CHESS.

T2WI was acquired with the following parameters: axial acquisition; repetition time (TR), 3200 ms; echo time (TE), 79 ms; flip angle (FA), excitation angle of 90°, refocusing angle of 120°; field of view (FOV), 185–199 × 220 mm; interpolation, off; phase resolution, 80%; partial Fourier, off; matrix, 378–406 × 448; 35 slices; parallel imaging factor, 2; voxel size, 0.5 × 0.5 × 3 mm; and acquisition time, 1 min 44–50 s.

### Image analysis: phantom

#### Analysis of image distortion

We measured the background signal (background, BG) of agarose gel on an image slice that had no artifact associated with the aneurysm clip. Signal higher than the average BG was considered to be signal pileup artifact, and signal lower than average BG was considered to be signal void artifact. The areas of artifact were measured as follows.

$${\text{Area}}\;{\text{of}}\;{\text{signal}}\;{\text{pileup}}\;{\text{artifact}} = {\text{signal}}\;{\text{higher}}\;{\text{than}}\;{1}.{5} \times {\text{average}}\;{\text{BG}}$$

$${\text{Area}}\;{\text{of}}\;{\text{signal}}\;{\text{void}}\;{\text{artifact}} = {\text{signal}}\;{\text{lower}}\;{\text{than}}0.{5} \times {\text{average}}\;{\text{BG}}$$

These data are provided in Supplementary Fig. 1.

### Image analysis: healthy volunteers

#### (a) Image quality assessment

The image quality of the trace-weighted diffusion images of each sequence type were qualitatively assessed for geometric distortion, susceptibility artifacts, overall image quality, and anatomic visualization of the trigeminal nerve and vestibulocochlear nerve using a 4-point Likert scale^20^. The criteria for image assessment are defined in Supplementary Table 1. The assessment was conducted by two board-certified neuroradiologists (A.S. and S.O. with 14 and 11 years of experience in neuroradiology, respectively). The three DWI sequences were provided in a random order, and each reader was blinded to the type of DWI sequence.

#### (b) Quantitative analysis

Distortion was examined quantitatively by measuring displacement between T2WI and each DWI sequence in the following eight brain regions: the frontal lobe near the frontal sinus and the anterior cranial base, parietal lobe, temporal tip, occipital lobe, pons, cerebellum near the mastoid antrum, and cerebellar hemisphere (Supplementary Fig. 2). Regions-of-interest (ROIs) were placed on four normal-appearing regions in the apparent diffusion coefficient (ADC) maps: the lateral ventricle, centrum semiovale, pons, and temporalis muscle^18^ (Supplementary Fig. 2). ROIs of the same size were then placed at the same locations in the three DWI sequences. Signal uniformity was quantified using coefficients of variation (ratio of the SD to the mean value) within each ROI^18^. ROI area was 95–110 mm^2^ in the lateral ventricle, centrum semiovale, and pons, and 10–15 mm^2^ in the temporalis muscle. Evaluation of distortion and the ROI measurements were performed by a board-certified radiologist (S.O. with 14 years of experience in neuroradiology) using ImageJ software version 1.53e (https://imagej.nih.gov/ij/), and approved by another board-certified radiologist (Y.F. with 23 years of experience in neuroradiology).

### Image analysis: patients after cerebral aneurysm clipping

#### (a) Image quality of the aneurysm clip

The area surrounding the aneurysm clip was qualitatively assessed for geometric distortion, susceptibility artifacts, and overall image quality using a 4-point Likert scale^20^. The criteria for image assessment are defined in Supplementary Table 2. The evaluation was conducted by the same two neuroradiologists who performed image analysis in the healthy volunteers.

#### (b) Analysis of clip-induced artifact

The length of clip-induced artifact was measured on T2WI and the trace-weighted image of each DWI sequence, and the difference in length between T2WI and each DWI was calculated. Distortion of cerebral parenchyma near the metal clip was evaluated when measuring the gap between T2WI and each DWI sequence. In the case that a patient had two clips, we evaluated the clip with more severe artifact. The measurement was performed using ImageJ software by the same neuroradiologist who performed image analysis in the healthy volunteers.

### Statistical analysis

Agreement between the image quality scores measured independently by the two radiologists was evaluated using weighted Cohen’s kappa coefficient^29^. Artifact length, coefficient of variation, and image quality score were compared among the three DWI sequences using the Friedman test. In the case of significant results, post-hoc analysis was performed using the Wilcoxon signed-rank test with Bonferroni correction. A *P* value less than 0.017 was considered statistically significant (Bonferroni adjustment).

Agreement among ADC values from the three DWI sequences was assessed by the intraclass correlation coefficient (ICC). The correlation coefficient was calculated to evaluate correlations of ADC values from the three DWI sequences, and Bland–Altman analysis was also performed. ADC values were compared among the three DWI sequences using one-way repeated measures analysis of variance (ANOVA) followed by a pairwise comparison with Bonferroni correction.

Statistical analysis was performed using Medcalc version 16.2 (MedCalc Software).

## Results

### Phantom study

**Figure 1:**
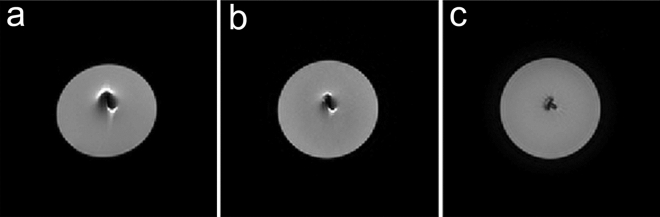
Images of an aneurysm clip embedded in agarose gel obtained by three DWI sequences. Image distortion around the clip is the largest in SS-EPI DWI (**a**), second largest in RESOLVE DWI (**b**), and the minimal in TGSE-BLADE DWI (**c**). Note the distorted contour of agarose gel in SS-EPI DWI and RESOLVE DWI.

Figure 1 shows images of the aneurysm clip embedded in agarose gel that were obtained by each DWI sequence. Image distortion around the clip was the greatest for SS-EPI DWI and the least for TGSE-BLADE DWI, which showed almost no distortion around the clip. RESOLVE DWI showed moderate distortion. The contour of the agarose gel was also distorted in SS-EPI DWI and RESOLVE DWI.

Areas of signal pileup artifact and signal void artifact are shown for the three DWIs in Supplementary Fig. 3. Area of signal void was the least in TGSE-BLADE DWI. There was no signal pileup only in TGSE-BLADE DWI.

### Participants

No participant was excluded from the study for the reason of motion artifact (Supplementary Fig. 4). Seventeen healthy volunteers (9 males, 8 females; mean age, 67.7 ± 11.6 years) and 20 patients (14 males, 6 females; mean age, 67.2 ± 11.2 years) were included. The demographics of all participants are shown in Supplementary Table 3.

### Healthy volunteers

#### Image quality

**Figure 2:**
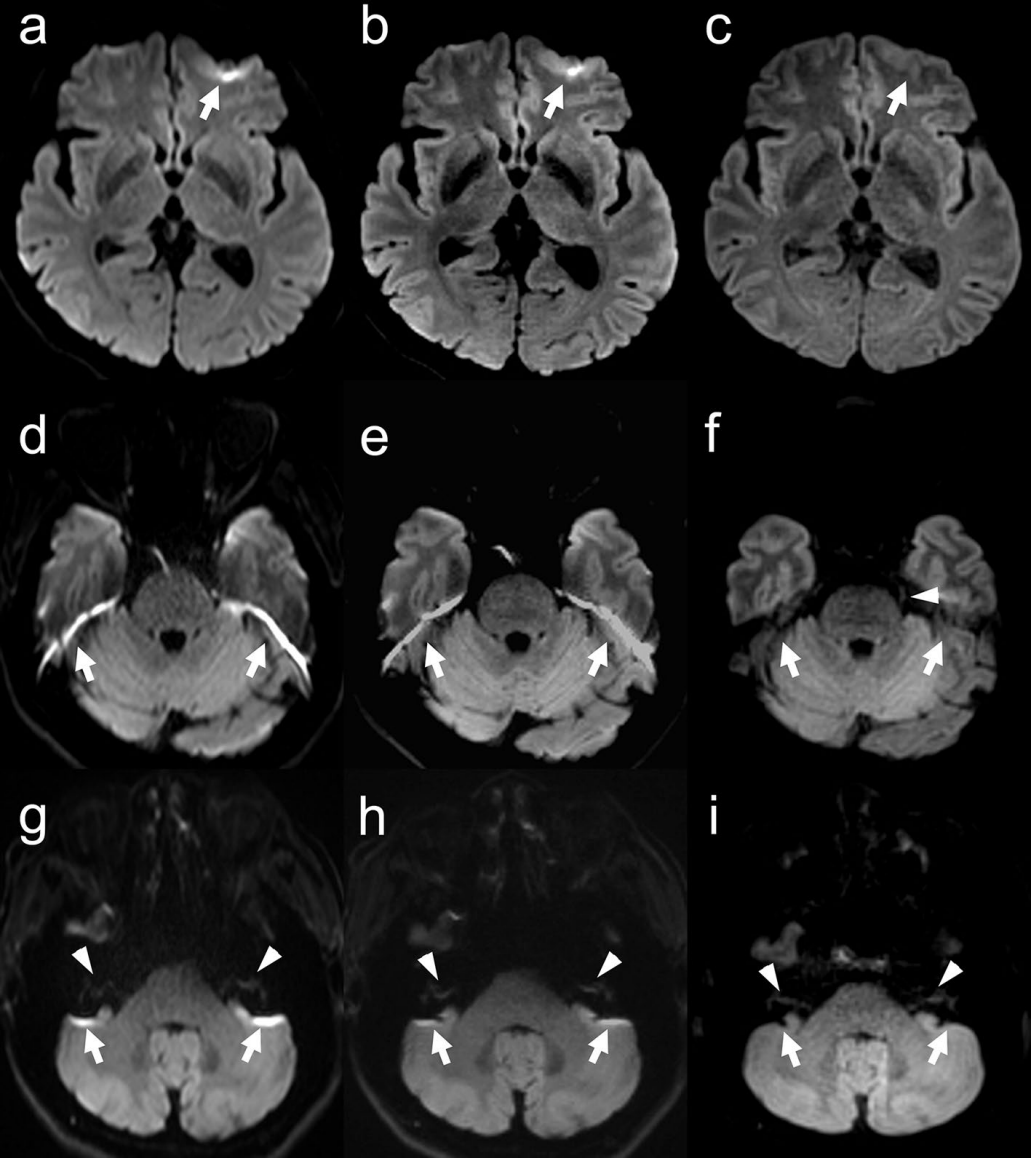
Representative images of SS-EPI DWI (**a**, **d**, **g**), RESOLVE DWI (**b**, **e**, **h**), and TGSE-BLADE DWI (**c**, **f**, **i**) in a 67-year-old healthy female volunteer. The arrows indicate susceptibility artifacts. Artifacts are seen near air–bone interfaces in RESOLVE DWI and SS-EPI DWI, whereas few artifacts are seen in TGSE-BLADE DWI. The trigeminal nerve (arrowhead) in visualized in TGSE-BLADE DWI (f) but is blurred in RESOLVE DWI and SS-EPI DWI. The vestibulocochlear nerve (arrowheads in g–i) are best visualized in TGSE-BLADE DWI.

Table 2 lists the image quality scores of readers 1 and 2 for each sequence. The kappa values of inter-rater agreement for geometric distortion, susceptibility artifacts, overall image quality, and anatomic visualization of trigeminal nerve and vestibulocochlear nerve were 0.98, 0.98, 0.89, 0.74 and 0.60, respectively, showing good or very good agreement. The scores for geometric distortion, susceptibility artifacts, and overall image quality were the best for both readers in TGSE-BLADE DWI (*P* < 0.001), and better in RESOLVE DWI than in SS-EPI (*P* < 0.001). The scores for anatomic visualization of the two cranial nerves were better for both readers in TGSE-BLADE DWI than in the other two DWI sequences (*P* < 0.001). There was no significant difference between RESOLVE DWI and SS-EPI DWI for the two cranial nerves in reader 1, and no significant difference only for trigeminal nerve in reader 2. Representative images of the three DWIs are shown in Fig. 2.

**Table 2:** Results of image quality for SS-EPI, RESOLVE DWI, and TGSE-BLADE DWI in healthy volunteers.

	SS-EPI DWI	RESOLVE DWI	TGSE-BLADE DWI	*P* value
**Reader 1**				
Geometric distortion	2.0 (2.0–2.0)	3.0 (3.0–3.0)	4.0 (4.0–4.0)	< 0.001
Susceptibility artifacts	2.0 (2.0–2.0)	3.0 (3.0–3.0)	4.0 (4.0–4.0)	< 0.001
Overall image quality	2.0 (2.0–2.0)	3.0 (3.0–3.0)	4.0 (4.0–4.0)	< 0.001
Visualization of trigeminal nerve	3.0 (2.0–4.0)	3.0 (2.8–3.0)	4.0 (4.0–4.0)	0.38^a^, < 0.001^b, c^
Visualization of vestibulocochlear nerve	3.0 (2.0–3.0)	3.0 (3.0–3.0)	4.0 (4.0–4.0)	0.16^a^, < 0.001^b, c^
**Reader 2**				
Geometric distortion	2.0 (2.0–2.0)	3.0 (3.0–3.0)	4.0 (4.0–4.0)	< 0.001
Susceptibility artifacts	2.0 (2.0–2.0)	3.0 (3.0–3.0)	4.0 (4.0–4.0)	< 0.001
Overall image quality	2.0 (2.0–2.0)	3.0 (3.0–3.0)	4.0 (4.0–4.0)	< 0.001
Visualization of trigeminal nerve	3.0 (2.0–3.0)	3.0 (3.0–3.0)	4.0 (4.0–4.0)	0.38^a^, < 0.001^b, c^
Visualization of vestibulocochlear nerve	2.0 (2.0–2.0)	3.0 (2.8–3.0)	4.0 (4.0–4.0)	< 0.001

Data are presented as the median (interquartile range) score. ^a^SS-EPI DWI versus RESOLVE DWI; ^b^RESOLVE DWI versus TGSE-BLADE DWI; ^c^SS-EPI DWI versus TGSE-BLADE DWI.

#### Image distortion

The lengths of distortion are shown in Table 3. Distortion was the lowest, with significance, in TGSE-BLADE DWI, and significantly lower in RESOLVE DWI than in SS-EPI DWI in the two parts of frontal lobe, temporal tip, cerebellum near the mastoid antrum, and pons (*P* < 0.001). There was no significant difference in distortion among the three DWI sequences in the occipital lobe or parietal lobe, or between SS-EPI and RESOLVE DWI in the cerebellar hemisphere.

**Table 3:** Comparison of the length of distortion (mm) among the three DWIs in healthy volunteers. Data are presented as the median (interquartile range) (mm).

	SS-EPI DWI	RESOLVE DWI	TGSE-BLADE DWI	*P* value
Frontal lobe near frontal sinus	3.44 (1.69–4.87)	1.60 (0.72–2.49)	0.49 (0.34–0.69)	< 0.001
Anterior cranial base	6.52 (5.01–8.96)	3.89 (2.98–5.41)	1.95 (1.22–2.48)	< 0.001
Parietal lobe	0.77 (0.49–0.82)	0.77 (0.49–1.00)	0.69 (0.45–0.97)	0.11^a^, 0.15^b^, 0.97^c^
Temporal tip	2.23 (1.58–3.22)	1.09 (0.94–1.89)	0.69 (0.48–0.88)	< 0.001
Occipital lobe	1.95 (0.94–2.91)	1.46 (0.98–2.56)	1.46 (0.98–1.96)	0.17^a^, 0.43^b^, 0.19^c^
Pons	5.73 (4.13–7.39)	2.98 (1.51–3.29)	0.46 (0.43–0.69)	< 0.001
Cerebellum near mastoid antrum	3.70 (2.93–7.69)	2.59 (1.55–4.94)	0.97 (0.71–1.59)	< 0.001
Cerebellar hemisphere	2.76 (1.15–4.61)	2.90 (0.92–3.90)	1.60 (0.63–2.81)	0.07^a^, < 0.001^b, c^

^a^SS-EPI DWI versus RESOLVE DWI; ^b^RESOLVE DWI versus TGSE-BLADE DWI; ^c^SS-EPI DWI versus TGSE-BLADE DWI.

ADC values of the three DWIs are shown in Supplementary Table 4. ICC among the ADC values of the three DWIs was 0.99 (95%CI 0.98–0.99). The correlation coefficient (r) for ADC values was 0.99 between SS-EPI DWI and TGSE-BLADE DWI, 0.99 between RESOLVE DWI and TGSE-BLADE DWI, and 1.00 between RESOLVE DWI and SS-EPI (Supplementary Fig. 5). Bland–Altman analysis was performed between the ADC measurements of SS-EPI DWI, RESOLVE DWI, and TGSE-BLADE DWI, and most data were distributed between ± 1.96 SD (Supplementary Fig. 6). Supplementary Table 5 summarizes the results of the coefficients of variation. Among the three sequences, signal inhomogeneity in the pons was lowest (*P* < 0.001) and signal inhomogeneity in the lateral ventricle was highest (*P* < 0.001) for TGSE-BLADE DWI.

### Patients after cerebral aneurysm clipping

#### Image quality

**Figure 3:**
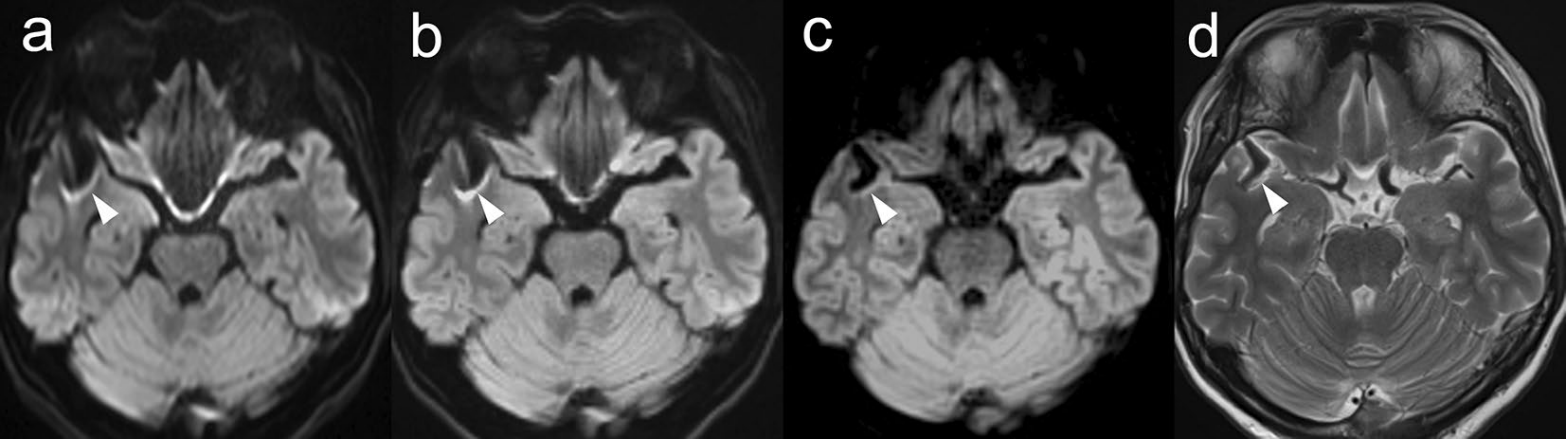
Representative images of SS-EPI DWI (**a**), RESOLVE DWI (**b**), TGSE-BLADE DWI (**c**) and T2WI (**d**) in a 60-year-old female patient after cerebral aneurysm clipping at the right middle cerebral artery. The arrowheads indicate the clip and the clip-induced artifacts. TGSE-BLADE DWI has the least artifact and distortion.

The image quality scores are listed in Table 4. The kappa values of inter-rater agreement were 0.78, 0.75, and 0.66 for geometric distortion, susceptibility artifacts, and overall image quality, respectively, showing good agreement. The scores for geometric distortion, susceptibility artifacts, and overall image quality were the best in TGSE-BLADE DWI, and better in RESOLVE DWI than in SS-EPI DWI (*P* < 0.001). Representative images of the three DWIs are shown in Figs. 3 and Supplementary Fig. 7.

**Table 4:** Results of image quality for SS-EPI DWI, RESOLVE DWI, and TGSE-BLADE DWI in patients who underwent cerebral aneurysm clipping.

	SS-EPI DWI	RESOLVE DWI	TGSE-BLADE DWI	*P* value
**Reader 1**				
Geometric distortion	2.0 (1.0–2.0)	3.0 (2.5–3.0)	4.0 (4.0–4.0)	< 0.001
Susceptibility artifacts	1.0 (1.0–2.0)	2.0 (2.0–3.0)	4.0 (4.0–4.0)	< 0.001
Overall image quality	1.5 (1.0–2.0)	3.0 (2.0–3.0)	4.0 (4.0–4.0)	< 0.001
**Reader 2**				
Geometric distortion	1.0 (1.0–2.0)	2.5 (2.0–3.0)	4.0 (4.0–4.0)	< 0.001
Susceptibility artifacts	1.0 (1.0–2.0)	2.5 (2.0–3.0)	4.0 (4.0–4.0)	< 0.001
Overall image quality	2.0 (2.0–2.0)	3.0 (3.0–3.0)	4.0 (4.0–4.0)	< 0.001

Data are presented as the median (interquartile range) score. ^a^SS-EPI DWI versus RESOLVE DWI; ^b^RESOLVE DWI versus TGSE-BLADE DWI; ^c^SS-EPI DWI versus TGSE-BLADE DWI.

#### Analysis of clip-induced artifact

Length of clip-induced artifact and distortion of the cerebral parenchyma near the metal clip were significantly the least in TGSE-BLADE DWI, and were significantly lower in RESOLVE DWI than in SS-EPI DWI (*P* < 0.01) (Table 5).

**Table 5:** Comparison of the length of clip-induced artifact and distortion of the cerebral parenchyma near the metal clip among the three DWIs.

	SS-EPI DWI	RESOLVE DWI	TGSE-BLADE DWI	*P* value
Clip-induced artifact	8.98 (4.64–11.36)	5.08 (3.39–7.62)	1.02 (0.26–3.19)	< 0.001
Distortion of the cerebral parenchyma near a metal clip	4.13 (2.48–5.95)	1.74 (0.97–2.92)	0.97 (0.69–1.63)	< 0.01^b^, < 0.001^a, c^

Data are presented as the median (interquartile range) (mm). Length of clip-induced artifact was the difference between T2WI and each DWI. ^a^SS-EPI DWI versus RESOLVE DWI; ^b^RESOLVE DWI versus TGSE-BLADE DWI; ^c^SS-EPI DWI versus TGSE-BLADE DWI.

## Discussion

This study demonstrated that TGSE-BLADE DWI had the best image quality, the least distortion, and the least susceptibility artifact compared with RESOLVE DWI and SS-EPI DWI, both in healthy volunteers and in patients after cerebral aneurysm clipping. RESOLVE DWI showed less image distortion and susceptibility artifacts than SS-EPI DWI, as reported previously^30,31^. TGSE-BLADE DWI had less image distortion and susceptibility artifacts than RESOLVE DWI, probably because distortion and T2* blurring effect are not completely removed in RESOLVE DWI due to the EPI readout scheme^25,32^.

Quantitative analysis of distortion by measuring displacement in images from T2WI and each DWI sequence showed that distortion was least near air–bone interfaces (e.g., frontal lobe, temporal tip, cerebellum, and pons) in TGSE-BLADE DWI, with significance. There was less difference in distortion between TGSE-BLADE DWI and the other DWI sequences with increasing distance from air–bone interfaces (e.g., occipital lobe and parietal lobe). Most previous studies have performed qualitative evaluation of distortion^20,23,24^. In the present study, however, we demonstrated quantitatively that distortion was reduced in intracranial regions. Furthermore, TGSE-BLADE DWI had the best qualitative score among the three DWI sequences in visualization of the trigeminal nerve and vestibulocochlear nerve. Our results suggest that TGSE-BLADE DWI is suitable for evaluating lesions near the paranasal sinus, mastoid cells, skull base, and cranial nerves.

ADC values for the centrum semiovale and pons were the highest in TGSE-BLADE DWI, and ADC values for the lateral ventricle and temporalis muscle were the highest in RESOLVE DWI, which might be caused by the timing of MPG application and TR^33^. The difference of ADC among 3 DWIs may be mainly caused by the differences of susceptibility artifacts appeared in each DWI. Susceptibility artifact in SS-EPI DWI and RESOLVE DWI was more severe than that in TGSE-BLADE DWI, which suggests that susceptibility artifacts affected calculation of ADC largely in SS-EPI DWI and RESOLVE DWI^24^. However, the ICC of ADC values among the three DWIs indicates an almost perfect agreement. Moreover, the correlation coefficients of ADC values among the three DWIs were 0.99–1.00, indicating very strong correlation. No previous research into TGSE-BLADE DWI has evaluated ADC values in different regions of normal cerebral parenchyma. Coefficients of variation of ADC values for the pons were the lowest in TGSE-BLADE DWI, and coefficients of variation of ADC values for the lateral ventricle were the highest in TGSE-BLADE DWI, which indicates that the signal for TGSE-BLADE DWI was more homogeneous in the pons and more inhomogeneous in the lateral ventricle compared with RESOLVE DWI and SS-EPI DWI. A previous study reported higher coefficients of variation in the lateral ventricle, centrum semiovale, and pons for PROPELLER DWI than for RESOLVE DWI and SS-EPI DWI^18^. These results indicate that signal homogeneity was greater for TGSE-BLADE DWI in the present study than for PROPELLER DWI in the prior study^18^, despite their similar acquisition times.

Clip-induced artifact and distortion of the cerebral parenchyma near the metal clip was the least in TGSE-BLADE DWI. In addition, the scores for geometric distortion, susceptibility artifacts and overall image quality were better in TGSE-BLADE DWI compared with other DWI sequences. Acute cerebral infarction occasionally occurs after cerebral aneurysm clipping^27,28^; however, small infarctions near the metal clip are not well depicted because of susceptibility artifacts, and it can be difficult to differentiate between acute infarction and susceptibility artifact. Our phantom study showed no signal pileup in TGSE-BLADE DWI, which reduces the likelihood of false positives in detecting acute infarction. Our results suggest that TGSE-BLADE DWI could be useful for identifying small areas of acute infarction and for differentiating infarctions from artifacts after aneurysm clipping.

There are several limitations in this study. First, the study included a small number of patients. Despite this limitation, image quality of the aneurysm clip was significantly the best, and image distortion was significantly the least in TGSE-BLADE DWI among all patients. Second, we performed the evaluation using only one type of aneurysm clip. Artifacts are observed most commonly at the ends, curved portion, and fenestrated portion of clips. In addition, various MR-conditional devices have been introduced recently, many of which will induce metallic artifact. It would be useful to perform low-artifact DWI in patients with such metallic devices. Third, we evaluated image quality and distortion only at 3 T MRI. Because the severity of image artifact has a non-linear relationship with field strength, image artifact should be evaluated at each field strength^34^. Fourth, we did not assess ADC values using a diffusion phantom. It is difficult to achieve good reproducibility of ADC measurements in a phantom study due to thermal control issues^35^. However, the primary focus of the present study was to compare distortion and artifacts among three DWIs that are used in clinical practice. Fifth, we did not assess signal-to-noise ratio (SNR). A previous study showed that SNR was slightly lower in TGSE-BLADE DWI than RESOLVE DWI^25^. This disadvantage of TGSE-BLADE DWI must be balanced against its ability to detect lesions near air–bone interfaces and metal clips, which is a strong advantage. Finally, the acquisition time in TGSE-BLADE DWI is clinically acceptable, but longer than that in SS-EPI DWI. To avoid SNR loss, we did not use any acceleration techniques (such as parallel imaging) in TGSE-BLADE DWI. In clinical practice, it might be worthwhile to utilize an appropriate acceleration factor that can balance acquisition time and SNR.

## Conclusion

TGSE-BLADE DWI had the best image quality among three DWI sequences in terms of distortion and artifacts, both in healthy volunteers and in patients who underwent aneurysm clipping. TGSE-BLADE DWI offers potential clinical advantages for detection of acute brain infarction in patients who have undergone aneurysm clipping.

## Supplementary Information

Supplementary Information.

## Data Availability

The datasets used and/or analyzed during the current study are available from the corresponding author on reasonable request. Software and versions used in the study: ImageJ software version 1.53e (https://imagej.nih.gov/ij/) and Medcalc version 16.2 (MedCalc Software).
