# Supplementary material for: Comparison of TGSE-BLADE DWI, RESOLVE DWI, and SS-EPI DWI in healthy volunteers and patients after cerebral aneurysm clipping
Source: Sci Rep. 2022 Oct 21;12:17689. doi: 10.1038/s41598-022-22760-6 (PMC9586944; doi:10.1038/s41598-022-22760-6)
Supplement: Supplementary file 1 — Supplementary Information. [file 41598_2022_22760_MOESM1_ESM.pdf]

Supplemental Materials

Supplementary Figure 1

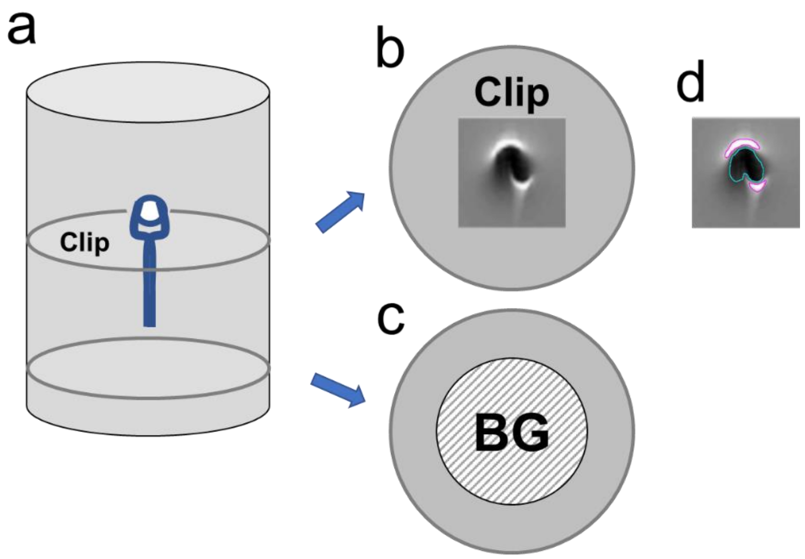

Image analysis method for the phantom. (a) This phantom was created by embedding one aneurysm clip in the center of a cylindrical container filled with agarose gel. (b) Image distortion around the clip was measured on the slice of clip. (c) Background signal (BG) was measured on a slice where no artifact was found. (d) The area of signal pileup (magenta) and signal void (cyan) were selected. Areas of "signal pileup artifact" and "signal void artifact" were calculated as follows.

Area of signal pileup artifact = Signal higher than 1.5×average BG.

Area of signal void artifact = Signal lower than 0.5×average BG.

Supplementary Figure 2

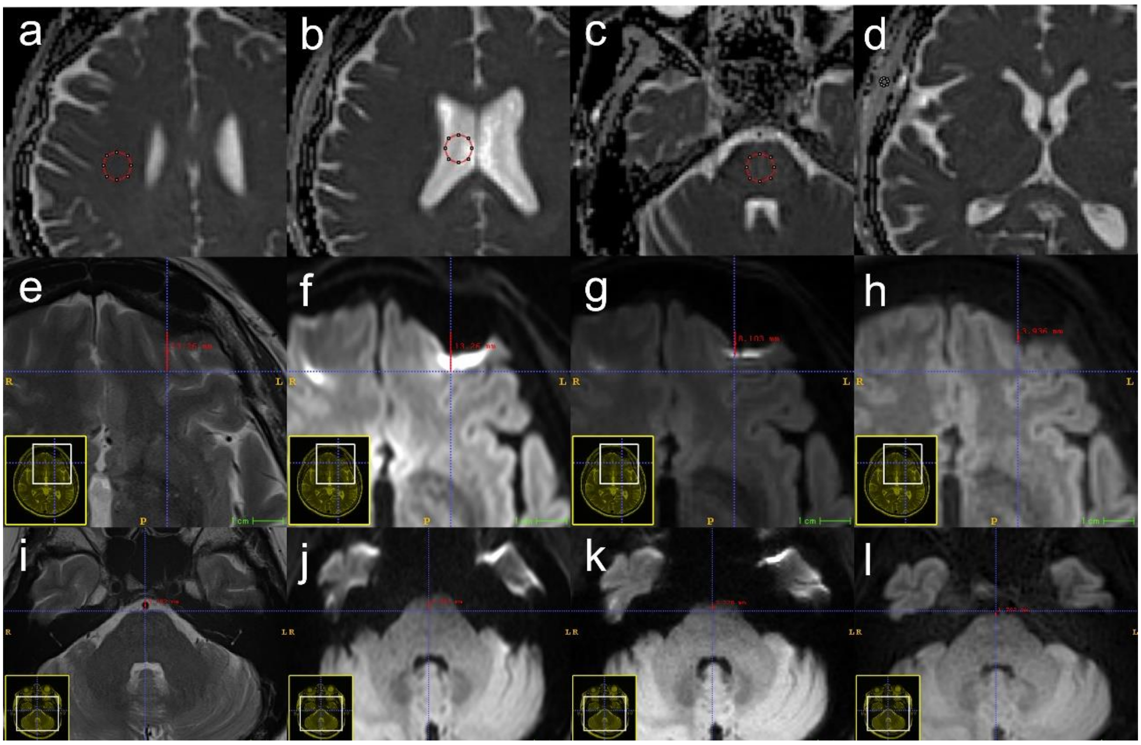

An example of regions-of-interest (ROIs) for the ADC measurements and lines for the distortion analysis. ROIs (red and white circles) were placed on four normal-appearing regions in the ADC maps: the centrum semiovale (a), lateral ventricle (b), pons (c), and temporalis muscle (d). Distortion (red line) was examined by measuring displacement between T2WI (e, i) and each DWI (f-h, j-l) in the frontal lobe near the frontal sinus (e-h) and pons (i-l). Distortion was the lowest in TGSE-BLADE DWI (h, i), and significantly lower in RESOLVE DWI (g, k) than in SS-EPI DWI (f, j).

Supplementary Figure 3

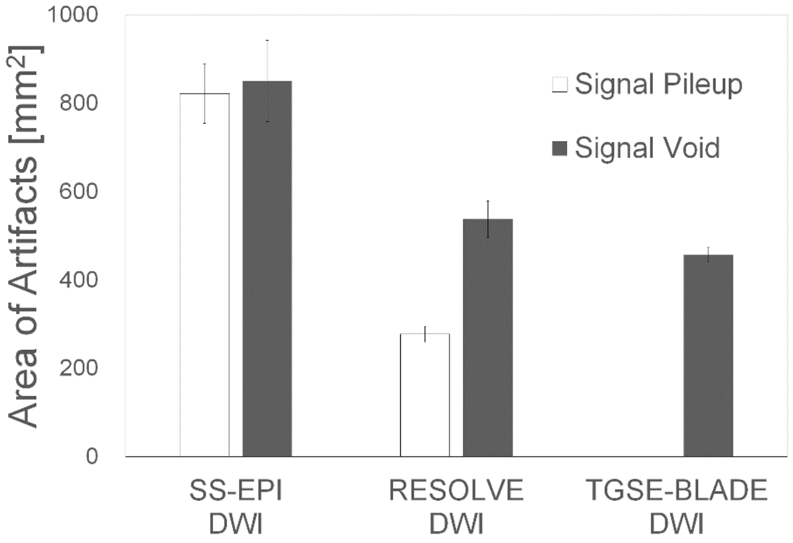

Areas of signal pileup artifact and signal void artifact in the three DWIs. Signal void is least in TGSE-BLADE DWI, and there is no signal pileup only in the TGSE-BLADE DWI sequence. RESOLVE DWI has the second lowest signal void but suffers from signal pileup.

Supplementary Figure 4

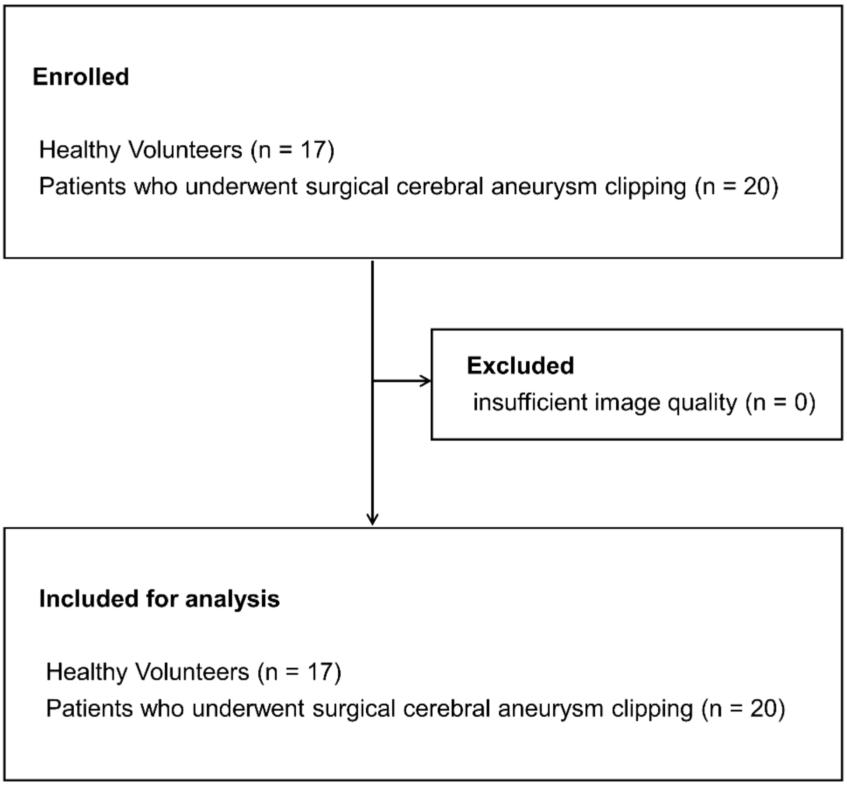

Flowchart of study enrollment.

Supplementary Figure 5

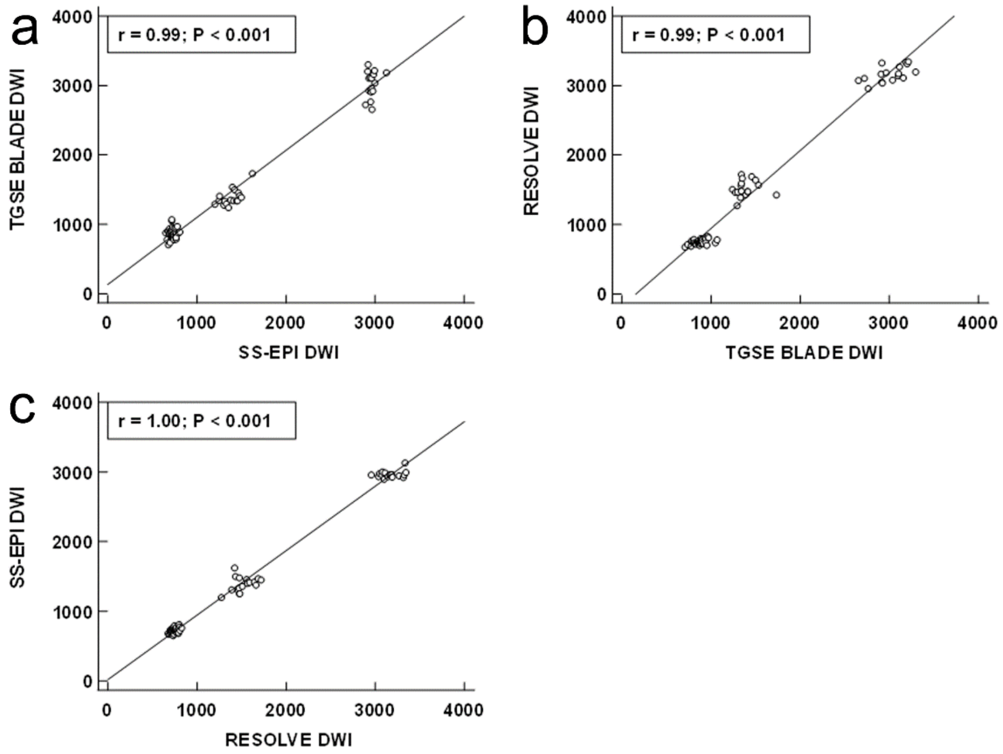

Scatter plots showing linear correlations of ADC values between (a) SS-EPI DWI and TGSE-BLADE DWI, (b) RESOLVE DWI and TGSE-BLADE DWI, and (c) RESOLVE DWI and SS-EPI DWI. All correlations were very strong.

Supplementary Figure 6

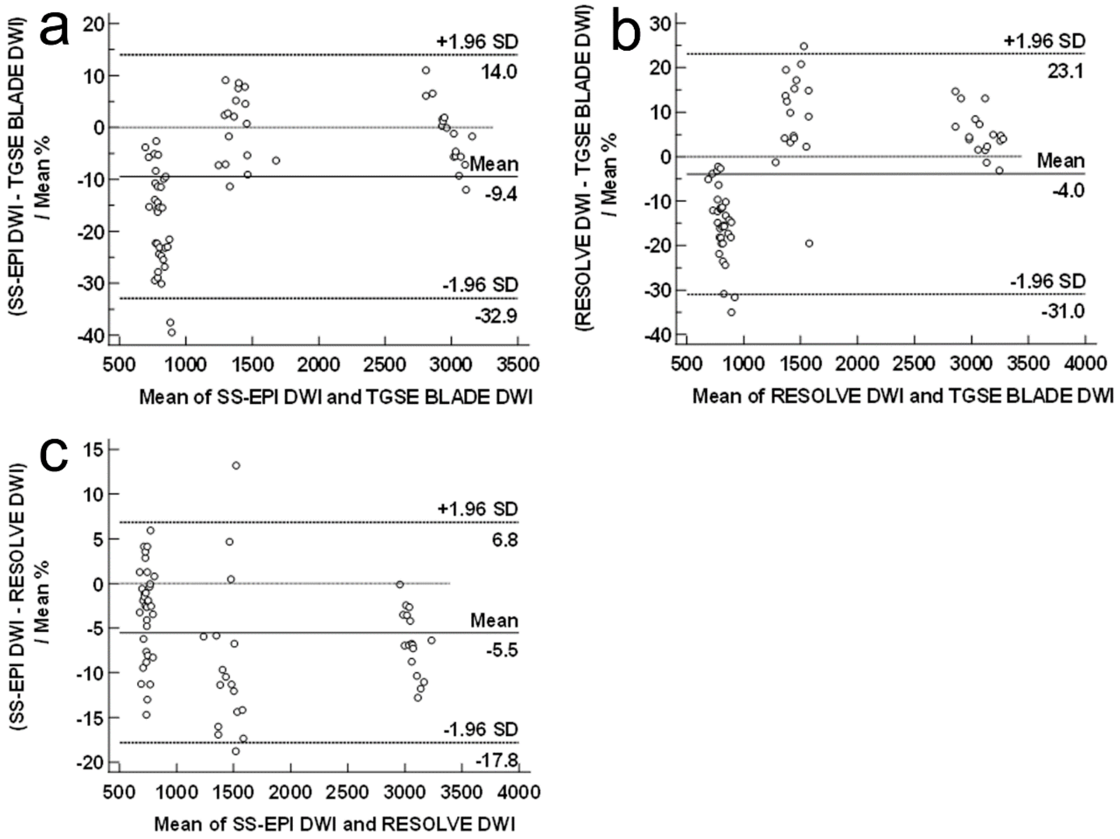

Bland–Altman analysis was performed between the ADC values of (a) SS-EPI DWI and TGSE-BLADE DWI, (b) RESOLVE DWI and TGSE-BLADE DWI, (c) RESOLVE DWI and SS-EPI DWI. Most data were distributed between ±1.96 SD.

Supplementary Figure 7

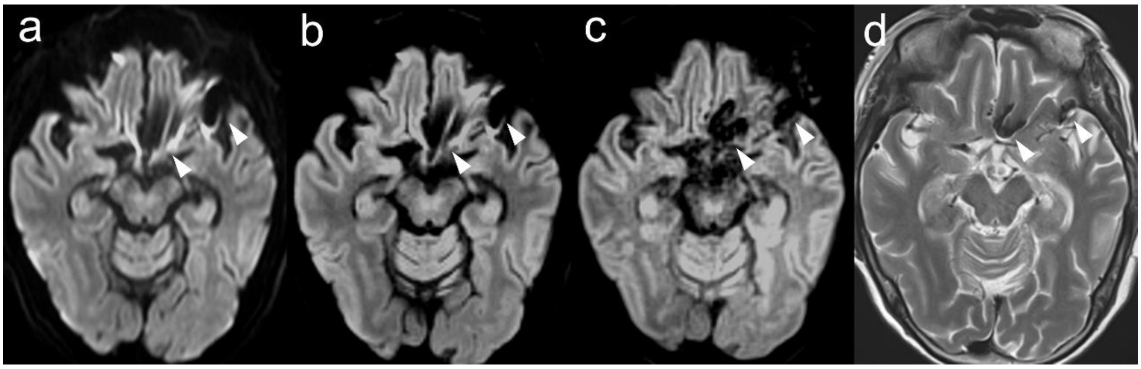

Representative images of SS-EPI DWI (a), RESOLVE DWI (b), TGSE-BLADE DWI (c) and T2WI (d) in a 76-year-old female patient after cerebral aneurysm clipping at the anterior communicating artery and left middle cerebral artery. The arrowheads indicate the clips and clip-induced artifacts. TGSE-BLADE DWI has the least artifact and distortion.

Supplementary Table 1

Criteria used for image assessment of trace-weighted DWI images in healthy volunteers.

	Geometric distortion	Susceptibility artifacts	Overall image quality	Visualization of the trigeminal nerve	Visualization of the vestibulocochlear nerve
1	Severe distortion	Severe artifacts	Poor, insufficient for diagnosis	Poor	Poor
2	Moderate distortion	Major artifacts	Fair, adequate for diagnosis	Acceptable	Acceptable
3	Mild distortion	Only minor artifacts	Good for diagnosis	Good	Good
4	No distortion	No artifacts	Excellent for diagnosis	Excellent	Excellent

Supplementary Table 2

Criteria used for image assessment of the area surrounding the aneurysm clip.

	Geometric distortion	Susceptibility artifacts	Overall image quality
1	Severe distortion	Severe artifacts	Poor, insufficient for diagnosis
2	Moderate distortion	Major artifacts	Fair, adequate for diagnosis
3	Mild distortion	Only minor artifacts	Good for diagnosis
4	No distortion	No artifacts	Excellent for diagnosis

Supplementary Table 3

Characteristics of volunteers and patients. Middle cerebral artery, MCA; internal carotid artery, ICA; anterior communicating artery, Acom; vertebral artery, VA; subarachnoid hemorrhage, SAH; arterial venous fistula, AVF.

Characteristic	Healthy volunteers (n = 17)	Patients after aneurysm clipping (n = 20)
Sex	9 males, 8 females	14 males, 6 females
Mean age ± SD (range)	67.7 ±11.6 (38–79)	67.2 ± 11.2 (40–86)
Location of aneurysmal clip	NA	MCA, n = 12<br>ICA, n = 4<br>Acom, n = 3<br>VA, n = 1
Past history of neurological disorder	NA	SAH, n = 4; subdural hematoma, ICA occlusion, cavernous hemangioma, pituitary adenoma, dural AVF, putaminal hemorrhage, Rathke's cleft cyst, all n = 1

Supplementary Table 4

ADC values in the lateral ventricle, centrum semiovale, pons, and temporalis muscle for SS-EPI, RESOLVE DWI, and TGSE-BLADE DWI. Data are presented as the mean ± SD (mm^2/s). [a]: SS-EPI DWI versus RESOLVE DWI; [b]: RESOLVE DWI versus TGSE-BLADE DWI; [c]: SS-EPI DWI versus TGSE-BLADE DWI.

	SS-EPI DWI	RESOLVE DWI	TGSE-BLADE DWI	*P* value
Centrum semiovale	728.8 ± 31.9	740.5 ± 35.9	825.7 ± 62.1	0.016[a], <0.001[b, c]
Lateral ventricle	2962.8 ± 51.0	3167.0 ± 116.5	3010.3 ± 184.9	<0.001[a], <0.01[b], 0.87[c]
Pons	723.5 ± 41.9	759.0 ± 36.0	923.5 ± 60.1	0.03[a], <0.001[b, c]
Temporalis muscle	1388.3 ± 106.5	1519.0 ± 117.0	1387.0 ± 118.1	<0.01[a, b], 1.0[c]

Supplementary Table 5

Comparison of the coefficients of variation of ADC values for SS-EPI DWI, RESOLVE DWI, and TGSE-BLADE DWI. Data are presented as the median (interquartile range). [a]: SS-EPI DWI versus RESOLVE DWI; [b]: RESOLVE DWI versus TGSE-BLADE DWI; [c]: SS-EPI DWI versus TGSE-BLADE DWI.

	SS-EPI DWI	RESOLVE DWI	TGSE-BLADE DWI	*P* value
Centrum semiovale	0.04 (0.03–0.05)	0.05 (0.04–0.06)	0.04 (0.04–0.05)	0.013[a], <0.01[b], 0.92[c]
Lateral ventricle	0.03 (0.03–0.04)	0.06 (0.05–0.06)	0.08 (0.07–0.10)	<0.001
Pons	0.13 (0.09–0.17)	0.11 (0.10–0.13)	0.09 (0.08–0.10)	0.39[a], <0.001[b, c]
Temporalis muscle	0.05 (0.05–0.07)	0.05 (0.04–0.06)	0.07 (0.05–0.08)	0.39[a], 0.12[b], 0.25[c]
